# Concomitant medication of psychoses in a lifetime perspective

**DOI:** 10.1002/hup.1209

**Published:** 2011-06-22

**Authors:** Maria Vares, Peter Saetre, Pontus Strålin, Sten Levander, Eva Lindström, Erik G Jönsson

**Affiliations:** 1 Department of Clinical Neuroscience, Karolinska Institutet and HospitalStockholm, Sweden; 2 Department of Health and Society, Malmö UniversityMalmö, Sweden; 3 Department of Forensic Psychiatry, Malmö University HospitalMalmö, Sweden

**Keywords:** antipsychotic drugs, concomitant medication, schizophrenia, retrospective, poly-pharmacy, lifetime

## Abstract

**Objective:**

Patients treated with antipsychotic drugs often receive concomitant psychotropic compounds. Few studies address this issue from a lifetime perspective. Here, an analysis is presented of the prescription pattern of such concomitant medication from the first contact with psychiatry until the last written note in the case history documents, in patients with a diagnosis of psychotic illness.

**Methods:**

A retrospective descriptive analysis of all case history data of 66 patients diagnosed with schizophrenia or schizophrenia-like psychotic disorders.

**Results:**

Benzodiazepines and benzodiazepine-related anxiolytic drugs had been prescribed to 95% of the patients, other anxiolytics, sedatives or hypnotic drugs to 61%, anti-parkinsonism drugs to 86%, and antidepressants to 56% of the patients. However, lifetime doses were small and most of the time patients had no concomitant medication. The prescribed lifetime dose of anti-parkinsonism drugs was associated with that of prescribed first-generation but not second-generation antipsychotics.

**Conclusions:**

Most psychosis patients are sometimes treated with concomitant drugs but mainly over short periods. Lifetime concomitant add-on medication at the individual patient level is variable and complex but not extensive. Copyright © 2011 John Wiley & Sons, Ltd.

## INTRODUCTION

Although antipsychotic drugs are regarded as a cornerstone in the treatment of schizophrenia and related psychoses, it is well known that a number of other drugs are also used in these patient categories. Concomitant medication, e.g. benzodiazepines, antidepressants, anti-parkinsonism drugs, mood stabilisers and beta-adrenergic blockers have received attention as add-on therapies or alternative drugs in the treatment of schizophrenia to manage certain specific as well as treatment resistant symptoms. This add-on medication give rise to potential disadvantages, such as pharmacokinetic and pharmacodynamic interactions, worsening of certain symptoms or risk for being continued without a need (Meyer, 2007). Concomitant medication indicating polypharmacy with two or more antipsychotic drugs is common and has been evaluated in several studies, including a previous report of the present subject material (Kontis *et al.*, 2010; Zink *et al.*, 2010; Jönsson *et al.*, 2011). The present report focuses on concomitant medication with non-antipsychotic drugs.

Benzodiazepines have, for many years broadly, been used in treatment of schizophrenia for sedation, as anxiolytics, in attempt to alleviate agitation, extrapyramidal side effects, aggressiveness and psychotic symptoms. Many studies have been conducted during the past four decades to evaluate usefulness and safety of benzodiazepines alone and in combination with antipsychotics in schizophrenia therapy. A review spanning studies performed during a period of 43 years could confirm some effectiveness of benzodiazepines only for short term sedation of acutely ill patients diagnosed with schizophrenia (Volz *et al.*, 2007). Another review verified therapeutic benefit of add-on benzodiazepines in the treatment of acute psychosis: patients receiving combination therapy had significantly less extrapyramidal symptoms compared with patients treated with only antipsychotics (Gillies *et al.*, 2005). There is also some evidence for a favourable effect of benzodiazepines in short time period treatment of akathisia (Lima *et al.*, 2002). Interest and research in benzodiazepines in the treatment of schizophrenia have abated after the introduction of second-generation antipsychotics (Stimmel, 1996). However, benzodiazepines are still relevant add-on medication in schizophrenia management. Lorazepam alone or in combination with first-generation antipsychotics given as intramuscular injection have been the treatment of choice for many years in treatment of acute agitation (Battaglia, 2005).

Very few studies reflect the role of non-benzodiazepine anxiolytics, sedatives and hypnotics in schizophrenia. Before the 1960s, barbiturates in combination with antipsychotics were commonly used in the treatment of psychoses as hypnotics, for sedation in therapy resistant cases (Prakash *et al.*, 1984) and for treatment of catatonic symptoms (Morrison, 1975). Barbiturates were gradually replaced by benzodiazepines once they became available (from the early 1960s). Some drugs from this heterogenous group of non-benzodiazepine sedatives, anxiolytics and hypnotics are abandoned in the treatment of psychoses, but others are still being used, like for instance the histamine H1 receptor antagonist, promethazine (Suzuki *et al.*, 2003; Huf *et al.*, 2005).

The occurrence of depressive states among patients with schizophrenia varies in different studies from 25% to 60% (Mallinger and Lamberti, 2007). Estimates of antidepressant prescription frequencies to patients with schizophrenia vary from 30% of inpatients to 43% of outpatients (Kasckow and Zisook, 2008). A recent review, investigating a broad range of antidepressive medication including tricyclic antidepressants, selective serotonin reuptake inhibitors (SSRIs), as well as other compounds in depression management of patients with schizophrenia reported week positive evidence for a therapeutic benefit (Micallef *et al.*, 2006).

A different focus in add-on medication with antidepressive drugs in schizophrenia is the treatment of negative symptoms. When compared with first-generation antipsychotic drugs, second-generation compounds are reported to be more efficient in reducing negative symptoms, but the advantages are small and variable (Möller, 2004). Studies investigating efficiency of add-on SSRI treatment of primary negative symptoms suggest that treatment is effective in chronic patients with schizophrenia when first-generation antipsychotics are combined with fluvoxamine or fluoxetine (Silver, 2001; Silver, 2004) and when SSRIs are used as adjunctive to second-generation compounds, such as clozapine and olanzapine (Silver *et al.*, 2009).

Acute extrapyramidal symptoms (EPS), such as akathisia, acute dystonia and Parkinsonism, are frequent and distressing side effects developing shortly after the start of antipsychotic treatment (Gray and Gournay, 2000). Long-term problems, e.g. tardive dyskinesia and akathisia, represent conditions which are very disturbing for patients with chronic schizophrenia (Kane, 2001). Akathisia has been associated with poor compliance and poor treatment outcome (Gerlach, 2002). The acknowledgement that EPS, at least to a certain degree, is a consequence of dopamine/acetylcholine imbalance secondary to dopamine blockade (Borison, 1983) resulted in common practice of treating akathisia and other EPS with anticholinergic drugs (Gray and Gournay, 2000). Two recent reviews, aiming to assess the efficacy of anticholinergic drugs for antipsychotic-induced acute akathisia and tardive dyskinesia, respectively, were not able to find any study meeting randomised clinical trial criteria. Consequently, no firm statement can be made regarding the efficacy of anticholinergic drugs in acute akathisia and tardive dyskinesia (Soares and McGrath, 2000; Rathbone and Soares-Weiser, 2006), with one exception: treatment with anticholinergic compounds appears to enhance the risk of tardive dyskinesia (Miller *et al.*, 2005).

Mood stabilisers like lithium and anti-epileptic drugs in combination with antipsychotic drugs have a well-known place in the clinical management of schizophrenia. Among the indications for the addition of mood stabilisers to antipsychotic agents in schizophrenia treatment are persistent problems with impulsivity and aggressiveness.

Lithium has been used in treatment of mood disorders for decades. Its role in schizophrenia treatment is still debated. A review examining whether augmentation of lithium to antipsychotic drugs in schizophrenia and related disorders is more effective than antipsychotic medication alone showed that patients who was given the combination had a significantly better treatment outcome. However, results became of borderline statistical significance when patients with schizoaffective disorder were excluded (Leucht *et al.*, 2007a).

Valproate is currently the most commonly used mood stabiliser in schizophrenia treatment (Citrome, 2009). Traditional indications for prescription of valproate are epilepsia and affective disorder. A recent review examining the function of valproate in combination with first-generation as well as second-generation antipsychotics concludes that there is no sufficient evidence supporting or refuting use of this drug in schizophrenia treatment (Schwarz *et al.*, 2008).

Carbamazepine is mainly used as an anticonvulsive agent and in the treatment of bipolar affective disorder. Carbamazepine is also an accepted alternative as mood stabiliser in schizophrenia treatment. Referring to currently existing results derived from randomised trials, carbamazepine cannot be recommended for routine clinical use in schizophrenia treatment, neither as a single substance nor in combination with antipsychotics (Leucht *et al.*, 2007b).

Another group of drugs having certain importance in treatment of patients with schizophrenia is cardiovascular agents. In the 1970s and early 1980s beta-adrenergic blocking drugs, such as propranolol, used in high doses, were supposed to be effective as alternative antipsychotics in neuroleptic resistant patients (Hayes and Schulz, 1983). It has also been suggested that beta-adrenergic antagonists are effective in the treatment of acute akathisia (Lipinski *et al.*, 1988; Kornischka *et al.*, 2007) and aggression associated with schizophrenia (Haspel, 1995).

Cardiovascular diseases are supposed to be the cause in more than 40% of all natural deaths in patients with schizophrenia (Koponen *et al.*, 2008). The most common cardiovascular side effect of antipsychotic drugs is orthostatic hypotension, which is common for some first-generation and second-generation antipsychotic compounds (Mackin, 2008). Another negative side effect of antipsychotics is their pro-arrhythmic potential, reflected in prolongated QT-interval, ventricular tachycardias and sometimes torsades de pointes: life-threatening ventricular tachyarrhythmia, associated with syncope and sudden death (Pacher and Kecskemeti, 2004). It has been reported that numerous first-generation (droperidol, thioridazine, chlorpromazine, haloperidol) and some second-generation agents (sertindole, clozapine and dose related effect in risperidone) are associated with prolongation of the QT-interval (Lindström *et al.*, 2005; Mackin, 2008).

Substance use disorder is widespread in patients with schizophrenia with a lifetime incidence almost three times higher than in the general population (Regier *et al.*, 1990). Co-occurring alcohol and/or drug addiction exacerbates long term prognosis in schizophrenia with increased relapse rates, more prominent positive symptoms (Swartz *et al.*, 2006), elevated risk for human immunodeficiency virus (HIV) (Rosenberg *et al.*, 2001) and homelessness (Caton *et al.*, 1994).

It has been suggested that persons with schizophrenia suffer from dopamine-mediated brain reward circuit dysfunction that is supposed to be a cause of co-occurring substance use disorder (Roth *et al.*, 2005; Green *et al.*, 2008). Although a recent literature search did not show any clear evidence for beneficial effects of traditional antipsychotics on co-occurring substance use disorder (Lubman and Berk, 2010), there is evidence that some second-generation antipsychotics, especially clozapine (Drake *et al.*, 2000; Zimmet *et al.*, 2000) can limit use of alcohol and other drugs in substance dependent patients with schizophrenia. Drugs that are commonly prescribed in alcohol dependency as disulfiram, naltrexone and acamprosate can be a useful component in treatment of this patient category (Petrakis *et al.*, 2006; Green *et al.*, 2008).

Increased prevalence of migraine among persons with psychiatric diagnoses has been reported by many studies (Low *et al.*, 2003; McIntyre *et al.*, 2006; Ortiz *et al.*, 2010). Common psychiatric comorbidity among migraine sufferers has also been reported (Breslau and Davis, 1993; Merikangas and Stevens, 1997; Ortiz *et al.*, 2010). Imbalance in serotonin (5-HT) neurotransmission is one of the possible factors linking pathophysiology of migraine (Hamel, 2007) with other disorders such as depression, anxiety, epilepsy and schizophrenia (Hedlund, 2009; Pytliak *et al.*, 2011). Sumatriptan, a 5-HT1 receptor agonist, traditionally used in migraine, modulates increased levels of brain serotonin that occurs during migraine attacks (Sakai *et al.*, 2008).

For migraine prevention, beta-adrenergic blockers (propranolol) (Linde and Rossnagel, 2004) and tricyclic antidepressants (amitriptyline, nortriptyline) (Punay and Couch, 2003) are commonly used (Galletti *et al.*, 2009). It has been reported that anti-epilepsy drugs (Zaremba *et al.*, 2006; Ettinger and Argoff, 2007) and second-generation antipsychotics, such as olanzapine (Silberstein *et al.*, 2002; Dusitanond and Young, 2009), can be useful in treatment of migraine.

We have previously analysed the use of antipsychotic drugs from a lifetime perspective in a sample of 66 patients with psychosis (Jönsson *et al.*, 2011). The aim of the present report was to investigate prescription patterns of non-antipsychotic drugs from a lifetime perspective in the same sample. This study has focus on the following topics: (i) add-on drugs, subdivided into broad categories, that are typically prescribed to psychosis patients and the total dose prescribed; (ii) the prescription patterns of add-on drugs in relation to diagnosis and disease course; (iii) possible associations between the prescribed amount of concomitant drugs and antipsychotics; (iv) whether the prescription of concomitant drugs is reasonably rational in clinical practice.

## METHODS

### Ethics

The study was approved by the Ethics Committee of the Karolinska Hospital, Stockholm, Sweden, and in accordance with the Declaration of Helsinki. All participating subjects gave informed consent.

### Patients

Data of the 66 patients (39 men and 27 women, mean age 41 years, range 24–62 years when recruited to the study) were analysed in the present report. All patients lived in Stockholm, Sweden, and were recruited to a study with a primary aim to analyse biological underpinnings of schizophrenia and related psychosis, such as molecular genetics and brain morphological variations (Jönsson *et al.*, 2006; Lawyer *et al.*, 2006). Subjects were treated in three out-patient clinics, specialised in the treatment of non-affective psychosis, in north-western Stockholm County. The patients diagnosed with schizophrenia or related psychoses by their treating psychiatrists were asked to participate. The study included a personal semi-structured interview, magnetic resonance imaging, donation of blood for DNA analysis, neuropsychological testing and permitting interviews with parents or siblings when available. For the present study, the first 66 patients, who accepted to release their medical records and for which these records had been obtained and read according to the procedure described below, were selected. The patients were diagnosed according to criteria of the Diagnostic and Statistical Manual for Mental disorders, third edition, revised (American Psychiatric Association, 1987) and fourth edition (American Psychiatric Association, 1995), on the basis of case history data and a clinical interview as previously described (Ekholm *et al.*, 2005; Vares *et al.*, 2006). We identified 48 patients with schizophrenia, eleven with schizoaffective disorder, one each with schizophreniform disorder, bipolar disorder and major depressive disorder, and four with a ‘psychosis not otherwise specified’ diagnosis. The patients were allocated to three groups: schizophrenia (*N* = 48), schizoaffective disorder (*N* = 11) and others (*N* = 7). The average time evaluated, i.e. from the first contact with psychiatry until the last note written in the medical record, varied from seven months to 40 years (median 14 years, lower quartile 5.6 years, upper quartile 21.5 years). This is summarised in Table 1.

**Table 1:** Sample characteristics. Mean and standard deviation are given for 66 patients divided by diagnosis

	Schizophrenia		Schizoaffective		Other	
			
	*N* = 48		*N* = 11		*N* = 7	
			
	(30 men, 18 women)		(4 men, 7 women)		(5 men, 2 women)	
Age (year)^a^	40.5	±8.9	43.1	±6.9	40.6	±15.1
Age of onset (year)	24.3	±5.4	28.2	±4.3	21.7	±4.1
Duration of illness (year)^a^	16.4	±9.7	14.6	±8.3	19.0	±14.7
Suicide attempts (*N*)	0.9	±1.1	0.4	±0.9	2.0	±1.5
Number of hospitalizations (*N*)	8.9	±10.3	12.2	±14.6	12.3	±17.9

a at year 2000

### Procedure

After giving informed consent, the patients underwent a clinical interview. During the interview, the patients were asked to give their consent on study-related access to their medical records from the psychiatric hospitals and outpatient clinics, which they had been in contact with. The obtained case history documentation was then scanned in order to identify eventual additional treatment units, which had been involved in the treatment of the patient. If so, medical records were requested also from these units. The general information about the patients comprised age of onset of illness, total number of hospitalisations, inpatient days and suicide attempts.

### Medication data

On the basis of this material, the life history of drug treatment was reconstructed. The medication history was subdivided into epochs of stable medication (same drugs, same doses). For each epoch, the following information was used: route of the drug (oral, injection, rectal, cutaneous), dosage per day (mg/d), ordination type (continuous, temporarily if needed), date of discontinuation. There were 1951 epochs available (spanning the years from 1960 to 2004) for analysis regarding the use of mood-stabilisers, antidepressants, anti-parkinsonism drugs, anxiolytics, sedatives and hypnotics, anti-migraine analgesics, drugs for the treatment of addiction and drugs for cardiovascular disorders. For the purpose of comparison doses were transformed to defined daily doses (DDD) equivalents (Table 2) (World Health Organization, 2007).

**Table 2:** Prescribed non-antipsychotic drugs, administration routes and defined daily doses

Drug category	Generic substance	ATC code	Adm route	DDD (mg)
A1 Mood stabiliser, lithium	Lithium	N05AN01	O	168
	Lithium carbonate	NA	O	2400
A2 Mood stabiliser, antiepileptics	Phenobarbital	N03AA02	O	100
			P	100
	Phenytoin	N03AB02	O	300
	Carbamazepine	N03AF01	O	1000
	Valproate	N03AG01	O	1500
B Antidepressants	Amitriptyline	N06AA09	O	75
	Buspirone	N05BE01	O	30
	Citalopram	N06AB04	O	20
	Fluoxetine	N06AB03	O	20
	Imipramine	N06AA02	O	100
	Clomipramine	N06AA04	O	100
	Maprotiline	N06AA21	O	100
	Mianserin	N06AX03	O	60
	Mirtazapine	N06AX11	O	30
	Moclobemide	N06AG02	O	300
	Nefazodone	N06AX06	O	400
	Nortriptyline	N06AA10	O	75
	Paroxetine	N06AB05	O	20
	Reboxetine	N06AX18	O	8
	Sertraline	N06AB06	O	50
	Trimipramine	N06AA06	O	150
	Venlafaxine	N06AX16	O	100
	Zimelidine	NA	O	200
C Anti-parkinsonism drugs	Biperiden	N04AA02	O	10
			P	10
	Orphenadrine	N04AB02	O	200
			P	200
	Trihexyphenidyl	N04AA01	O	10
D1 Anxiolytics, benzodiazepine derivatives	Alprazolam	N05BA12	O	1
	Chlordiazepoxide	N05BA02	O	30
	Diazepam	N05BA01	O	10
			P	10
			R	10
	Flunitrazepam	N05CD03	O	1
	Clonazepam	N03AE01	O	8
			P	8
	Chlordiazepoxide	N05BA02	O	30
	Lorazepam	N04AA01	O	2.5
	Nitrazepam	N05CD02	O	5
	Oxazepam	N05BA04	O	50
	Zolpidem	N05CF02	O	10
	Zopiclone	N05CF01	O	7.5
D2 Other anxiolytics, hypnotics and sedatives	Aprobarbital	N05CA05	O	100
			P	100
	Glutethimide	N05CE01	O	250
	Hexapropymate	N05CM10	O	400
	Hydroxyzine	N05BB01	O	75
	Clomethiazole	N05CM02	O	1500
	Chloral	N05CC01	O	1000
	Amobarbital	N05CA02	O	100
	Prochlorperazine	N05AB04	O	100
	Promethazine	R06AD52	O	25
			P	25
			R	25
	Propiomazine	N05CM06	O	25
	Melantonin	N05CH01	O	2
E3 Anti-migraine analgesics	Ergotamine	N02CA72	O	4
	Sumatriptan	N02CC01	O	50
F1 Drugs for treatment of addiction	Disulfiram	N07BB01	O	200
	Kalciumkarbamid	V03AA02	O	50
G Drugs for cardiovascular disorders	Amiloride	C03DB01	O	10
	Hydrochlorothiazide	C03EA01	O	25
	Bendroflumethiazide	C03AB01	O	2.5
	Dihydroergotamine	N02CA01	O	4
	Etilefrine	C01CA01	O	50
	Furosemide	C03CA01	O	40
	Gemfibrozil	C10AB04	O	1200
	Losartan	C09CA01	O	50
	Metoprolol	C07AA05	O	150
	Propranolol	C07AB02	O	160
	Simvastatin	C10AA01	O	30

Adm route, administration route; DDD, defined daily doses; O, oral; P, parenteral; R, rectal.

### Statistical analysis

Summary descriptions of the data and statistical analysis were carried using the sas software (sas/stat® software, version 9.01, SAS institute Inc., Cary, NC).

#### Disease course and diagnostic categories

Prior to any statistical analysis, the correlation structure of three variables describing course, which to some extent mirror aspects of severity of the disease state, was explored with a principal component analysis. The variables analysed were age of onset, number of suicide attempts and number of hospitalisations. To make the last two variables independent of the observation period, these variables were regressed against the time elapsed since the first psychotic episode (log transformed). Then, the residuals from the regression were used as time normalised variables (for details, see (Jönsson *et al.*, 2011)). We used the log-transformed scores along the first principal component as an index summarising the disease course of the individual patients.

To characterise whether the type and amount of prescribed medications varied with diagnosis or disease course, a series of statistical tests were carried out. For the purpose of the analysis, diagnosis was split into three categories: *schizophrenia* (*N* = 48), *schizoaffective disorder* (*N* = 11) and *other* psychotic diagnosis (*N* = 7). The latter category included schizophreniform, bipolar and major depressive disorders as well as ‘psychosis not otherwise specified’.

#### Analysis of drug prescription

We analysed the prescription of the following broad categories: mood stabiliser, lithium (A1); mood stabiliser, anti-epileptics (A2); antidepressants (B); Anti-parkinsonism drugs (C); anxiolytics, benzodiazepine derivatives (D1); other anxiolytics, hypnotics and sedatives (D2); anti-migraine analgesics (E3); drugs for treatment of addiction (F1); and drugs for cardiovascular disorders (G).

The prescription of drugs within these categories was analysed in two steps: First, we used a logistic regression (Proc Genmod) to test whether the prescription of drugs within each category at least once (1/0) varied with diagnosis or disease course. Next, we analysed whether the total DDD (log transformed) of prescribed drugs varied with diagnosis or disease course, using time since onset as a covariate (log transformed) (Proc GLM, SAS v. 9.1). The second analysis was restricted to four categories (antidepressants (B), anti-parkinsonism drugs (C), anxiolytics, hypnotics and sedatives (D1, D2)), which were prescribed to at least 50% of patients. Finally, we examined the relationship between the prescribed amount of drugs within these four categories and the prescribed amount of antipsychotics. In this analysis, the total DDD (log-transformed) within each of the four drug categories was modelled as a linear function of the total DDD of antipsychotics, accounting for the effects of diagnosis and disease course by including these as additional factors in the statistical model.

## RESULTS

### Concomitant drugs prescribed to psychotic patients

**Figure 1:**
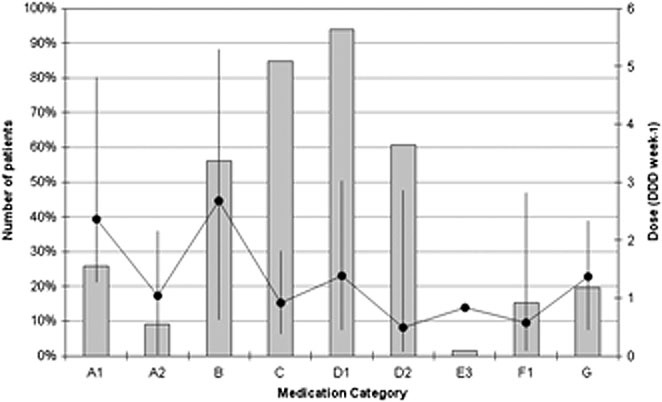
Prescription of non-neuroleptic medication to 66 patients with schizophrenia. Grey bars represent number of patients (*N*_total_ = 66). Black circles represent the typical prescribed dose (median) with whiskers describing the 50% range (1st–3rd quartile). A1, Mood stabiliser, lithium; A2, Mood stabiliser, anti-epileptics; B, Antidepressants; C, Anti-parkinsonism drugs; D1, Anxiolytics, Benzodiazepine derivatives; D2, Other anxiolytics, hypnotics and sedatives; E3, Anti-migraine analgesics; F1, Drugs for treatment of addiction; G, Drugs for cardiovascular disorders

Prescription records of concomitant medication that fell into nine broad categories were analysed in this study. The most frequently prescribed categories of medication were anxiolytics, antidepressants and medication against Parkinsonism (Figure 1). That is, benzodiazepines and benzodiazepine-related anxiolytic drugs had been prescribed to 95% (*N* = 63) of the patients, other anxiolytics, sedatives or hypnotic drugs to 61% (*N* = 40), anti-parkinsonism drugs to 86% (*N* = 57) and antidepressants to 56% (*N* = 37) of the patients. We also noted that lithium or anti-epileptic drugs, often used as mood-stabilisers, and drugs for the treatment of addiction had been prescribed to 26% (*N* = 17), 9% (*N* = 6) and 15% (*N* = 10) of the patients, respectively. The proportion of patients that had been prescribed medication from the other broad categories listed varied from 20% (*N* = 13) to 1.5% (*N* = 1) (Figure 1).

The typical lifetime dose prescribed to a patient within each of these categories was calculated as the cumulative dose at the end of the study, divided by the time since the first prescription within the category. For most medication categories, this dose was approximately one DDD per week of calendar time, with the exception of lithium and antidepressants were the prescribed dose was approximately twice as large. The fact that the prescribed doses from a lifetime perspective generally were low was due to the limited time periods that these drugs were prescribed to each patient. When the drugs were prescribed, the doses were close to one DDD (data not shown). The average number of different generic substances prescribed to patients within a category rarely exceeded two. The exception to this rule was benzodiazepines and benzodiazepine-related anxiolytic drugs. This category averaged three different substances prescribed per patient.

### Variation in prescription patterns with diagnosis and course of the disease

The prescription pattern in relation to diagnosis and course is presented in Table 3. In total, 17 patients had been prescribed lithium. As expected, the prescription varied significantly with diagnostic category (*p* < 0.0001), but also with disease course (*p* < 0.001). Nine of the 11 patients diagnosed with schizoaffective disorder (82%) had been prescribed lithium, whereas the corresponding percentage was 13% and 17% for individuals with schizophrenia or other diagnoses. The prescription of lithium to patients who were not diagnosed with schizoaffective disorder depended on the course of the disorder: no patient in the least affected quartile had been prescribed lithium, whereas the corresponding proportion of patients were 8% and 13% for the second and third quartile, and 36% for the most severely affected quartile.

**Table 3:** Prescription of non-antipsychotic medication to 66 patients, divided with respect to diagnosis (SCZ, schizophrenia; SCA, schizoaffective disorder) and disease course (Q1–Q4, quartiles of progressively worsening course). Number of patients prescribed agents from each drug category is listed together with the typical (median) prescribed dose, expressed in defined daily doses per week (within brackets)

		Diagnosis			Disease course			
			
Drug category		SCZ (*N* = 48)	SCA (*N* = 11)	Other (*N* = 7)	Q1 (*N* = 16)	Q2 (*N* = 17)	Q3 (*N* = 17)	Q4 (*N* = 16)
A1	Mood stabiliser, lithium	6 (3.1)	9 (2.4)	2 (1.5)	2 (4.0)	5 (1.5)	3 (5.2)	7 (2.5)
A2	Mood stabiliser, antiepileptics	4 (1.3)	1 (1.7)		1 (11.7)	2 (1.0)		2 (1.1)
B	Antidepressants	26 (2.5)	4 (2.7)	7 (4.7)	11 (4.4)	5 (1.1)	11 (4.3)	10 (2.4)
C	Anti-parkinsonism drugs	41 (0.9)	8 (0.6)	6 (1.3)	9 (0.4)	15 (0.9)	15 (0.7)	16 (1.1)
D1	Anxiolytics, benzodiazepine derivatives	44 (1.4)	11 (0.8)	7 (2.9)	15 (0.7)	16 (1.5)	16 (1.4)	15 (2.9)
D2	Other anxiolytics, hypnotics and sedative	29 (0.9)	8 (0.1)	3 (1.1)	8 (0.5)	10 (0.5)	9 (0.02)	13 (1.0)
E3	Anti-migraine analgesics			1 (0.8)				1 (0.8)
F1	Drugs for treatment of addiction	9 (0.7)		1 (0.1)	2 (2.8)	1 (0.03)	3 (2.6)	4 (0.4)
G	Drugs for cardiovascular disorders	9 (1.4)	2 (0.3)	2 (2.3)	2 (0.9)	3 (4.7)	5 (0.6)	3 (2.3)

Thirty-seven patients (53%) had been prescribed antidepressants at least once, and prescription varied significantly with diagnosis (*p* = 0.004), but not with disease course (*p* = 0.69). All patients in the *Other* diagnostic group (*n* = 7) had been prescribed anti-depressive drugs, whereas the percentage of schizoaffective and patients with schizophrenia who were prescribed anti-depressives was 36 and 54%, respectively.

Fifty-seven patients (86%) had been prescribed anti-parkinsonism drugs, and the frequency was similar across the three diagnostic categories (*p* = 0.54). Disease course significantly affected the fraction prescribed (*p* < 0.01): among the quartile of least affected individuals only 56% had been prescribed anti-parkinsonism drugs. The corresponding percentage for the other three quartiles varied between 88% and 100%. In addition, the longer period of time a patient had been affected with psychotic symptoms, the more likely it was that they had been prescribed anti-parkinsonism drugs. For the remaining drug categories, neither diagnostic category or disease course, nor time since onset explained any significant variation in prescription patterns.

The lifetime dose of anxiolytics varied with diagnosis and disease course (factoring out the time since the first prescription). The lifetime prescription of benzodiazepine derivatives tended to increase with disease course (*p* < 0.05), and the average amount prescribed to patients within the quartile of worst affected patients was four times as large as that prescribed to patients in the least affected quartile. In addition, patients diagnosed with schizoaffective disorder were prescribed on average only 10% of the amounts of other anxiolytics, hypnotics and sedatives (E2) as compared with patients diagnosed with schizophrenia (*p* < 0.05).

Of the four commonly prescribed concomitant drug categories, only the lifetime dose of anti-parkinsonism drugs was associated with that of prescribed antipsychotics (*p* < 0.001). On average, one DDD unit anti-parkinsonism medication was prescribed per seven DDD units of antipsychotics, and this relationship did not vary significantly with the lifetime prescription of antipsychotics, with diagnosis or with disease course. The relationship was primarily driven by the prescribed dose of first-generation antipsychotics (*p* < 0.001), and the dose of second-generation antipsychotics did not explain any significant variation of anti-parkinsonism medication when the effect of first-generation drugs was accounted for in the analysis (*p* = 0.34).

## DISCUSSION

There are a number of various long-term studies regarding antipsychotic treatment in schizophrenia, which evaluate also aspects of add-on medication (Williams *et al.*, 1999; Luo *et al.*, 2002; Glick *et al.*, 2004; Edlinger *et al.*, 2005; Haro and Salvador-Carulla, 2006). To the best of our knowledge, the present report is the only real-life study describing lifetime prescription patterns of concomitant drugs in the treatment of schizophrenia and related disorders. The main findings were (i) that almost all patients at some point had been prescribed concomitant medication; (ii) in contrast to antipsychotic drugs, these treatment periods were short, corresponding to 14% of the total time of antipsychotic medication, with one exception, anti-parkinsonism drugs (28%); (iii) in contrast to antipsychotic agents (typically a patient had been prescribed seven different compounds), prescription of concomitant drugs were limited to much fewer compounds; (iv) the most frequently used concomitant drugs matched the most common unmet needs of patients with a psychotic illness: anxiety, sleeping problems, affective symptoms, side effects; (v) there were small variations in the prescription pattern of concomitant medication with respect to diagnosis and disease course; and (vi) the prescription of anti-parkinsonism drugs was associated with that of prescribed first-generation but not second-generation antipsychotics. Summing up, the prescribing clinicians appear to have acted rationally and with caution when selecting concomitant medication. The time-limited nature of the prescriptions suggests that the prescriptions were motivated by specific transient problems. In contrast, the same clinicians changed much more when prescribing antipsychotic drugs, probably because no antipsychotic drug actually works well enough taken therapeutic and side effects into account (Jönsson *et al.*, 2011).

The present study examined the patients from a small catchment area with a well developed psychiatric service. Although the sample size was limited, the results in general agree well with the previous findings. For example, in the large prospective Schizophrenia Outpatient Health Outcomes study, the most frequently prescribed adjunctive medication to patients treated with antipsychotics were anxiolytics/hypnotics (22%–37% of patients), anticholinergics (5%–29%), antidepressants (8%–23%) and mood stabilisers (7%–19%) (Novick *et al.*, 2005). Another similar study of 456 patients with schizophrenia in the USA reported that 37% of patients received antidepressants, 33% mood stabilisers and 23% anxiolytic drugs (Mallinger and Lamberti, 2007). None of these above cited studies examined patients in a lifetime perspective, and thus the higher fraction of patients receiving concomitant medication in our material is likely to be an effect of a wider observation window associated with a higher probability that particular symptoms requiring add-on medication occur in each individual patient. Another potential origin of elevated frequencies in concomitant medication could be caused by different drug availability and changes in prescription policies with time. The usage of novel antipsychotic drugs is reported to be associated with less need of add-on medications (Pierre, 2005; Haro and Salvador-Carulla, 2006). A considerable number of patients in our material initiated their treatment before the second-generation compounds existed as therapeutic alternative. At present, second-generation antipsychotic drugs, e.g. aripiprazole, olanzapine, quetiapine, risperidone and ziprasidone, are suggested as mono-therapies for both schizophrenia and bipolar disorder because of their high antipsychotic potential and favourable extrapyramidal side effect profile (Citrome *et al.*, 2005).

Our findings that lithium was primarily prescribed to schizoaffective patients, and that lithium rarely is prescribed to patients with schizophrenia with comparatively mild symptoms are in accordance with the literature were lithium primarily is prescribed to patients with affective symptoms (Shorter, 2009). Add-on lithium therapy in schizophrenia is relatively unfrequent, and primarily used in patients resistant to long term antipsychotic treatment and patients who need long and repetitive hospitalisations (Leucht *et al.*, 2007a).

In the present study, all patients that were not diagnosed with schizophrenia or schizoaffective disorder were prescribed antidepressant drugs, whereas the prescription to patients diagnosed with schizophrenia and schizoaffective disorder were more restrictive. These findings are in line with the literature as the risk of antidepressants to exacerbate positive symptoms makes clinicians more cautious with respect to core patients with schizophrenia. Among psychiatrists who prescribed combination therapy, the most frequent combination was SSRI added to a novel antipsychotic (Addington *et al.*, 2002).

Even if second-generation antipsychotics have milder EPS side effects, such side effects appear with high doses (Kane, 2001). Patients with therapy-resistant schizophrenia are generally prescribed maximal antipsychotic doses, which could at least to a certain extent, explain why usage of anti-parkinsonism medication was so high in patients with the most troublesome course.

A Swedish study reported that the lifetime prevalence of alcohol and substance abuse in a sample of patients with schizophrenia was 48% (Cantor-Graae *et al.*, 2001). In the present study, 10 patients (15%) were prescribed treatment for addiction. Only two substances, both facilitating the accumulation of acetaldehyde and activation of an intolerability reaction, had been prescribed to prevent alcohol use of the patients in the present study. This may mirror that several of the patients had been ill for a long period of time and that no other specific anti-alcohol pharmacological treatments were available before the 1990s. Also, the use of anti-craving agents in the treatment of alcohol dependence has been scarcely prescribed in Sweden, and it has been argued for a more widespread use of these drugs.

In a Swedish study spanning the years 1984–2009, the prescription pattern of antihypertensive and lipid lowering drugs was analysed. Six per cent to 13% and 0.3% to 9% of the individuals aged from 25 to 64 years were prescribed drugs for hypertension and hyperlipidemia, respectively, whereas the prescription rate raised several-fold in higher age groups (Eriksson *et al.*, 2011). This suggests a roughly similar prescription frequency of drugs for cardiovascular disorders in the patients of the present study (20%) as in the general population.

The participating patients took part in demanding biological research and signing an informed consent declaration was needed to be included. This is likely to violate the representation of the general psychosis population. In addition, the retrospective design, the small number of participants and the confinement to a small catchment area with well-developed psychiatric services motivate caution in interpretation of the results, particularly, with respect to generalisation to other contexts and countries. Too little research is invested in how clinicians actually select and motivate prescriptions and it appears to be many unknown factors in this process (Levander *et al.*, 2007).

## CONCLUSION

Lifetime concomitant add-on medication at the individual patient level is variable and complex but not extensive. During the present long-time investigation with a lifetime perspective, almost all patients were prescribed anxiolytic drugs, the vast majority anti-parkinsonism drugs and more than half antidepressants, however mostly during limited time periods. The prescribed lifetime dose of anti-parkinsonism drugs was associated with that of prescribed first-generation antipsychotics. Given the limited sample size and restricted catchment area, the present results may not be generalised to the psychosis population in general.

## CONFLICT OF INTEREST

AstraZeneca partly financed the present study. However, neither AstraZeneca, nor any of the other sponsors had any role in the study design, in the collection, analysis or interpretation of data, in the writing of the report and in the decision to submit the report for publication with the following exceptions: AstraZeneca supported the study in a late phase of the project on the premises that a report should be submitted. Officials at AstraZeneca Sweden also read and suggested proposals for minor changes in the first draft of the manuscript. All statistical analyses were performed by PS (peter.saetre@ki.se) at the Department of Clinical Neuroscience, Karolinska Institutet. EGJ has served as an unpaid consultant for Eli-Lilly.

## References

[b1] Addington DD, Azorin JM, Falloon IR, Gerlach J, Hirsch SR, Siris SG (2002). Clinical issues related to depression in schizophrenia: an international survey of psychiatrists. Acta Psychiatr Scand.

[b2] American Psychiatric Association (1987). Diagnostic and Statistical Manual of Mental Disorders, 3rd edn-Revised.

[b3] American Psychiatric Association (1995). Diagnostic and Statistical Manual of Mental Disorders, 4th edn, International Version.

[b4] Battaglia J (2005). Pharmacological management of acute agitation. Drugs.

[b5] Borison RL (1983). Amantadine in the management of extrapyramidal side effects. Clin Neuropharmacol.

[b6] Breslau N, Davis GC (1993). Migraine, physical health and psychiatric disorder: a prospective epidemiologic study in young adults. J Psychiatr Res.

[b7] Cantor-Graae E, Nordstrom LG, McNeil TF (2001). Substance abuse in schizophrenia: a review of the literature and a study of correlates in Sweden. Schizophr Res.

[b8] Caton CL, Shrout PE, Eagle PF, Opler LA, Felix A (1994). Correlates of codisorders in homeless and never homeless indigent schizophrenic men. Psychol Med.

[b9] Citrome L (2009). Adjunctive lithium and anticonvulsants for the treatment of schizophrenia: what is the evidence?. Expert Rev Neurother.

[b10] Citrome L, Goldberg JF, Stahl SM (2005). Toward convergence in the medication treatment of bipolar disorder and schizophrenia. Harv Rev Psychiatry.

[b11] Drake RE, Xie H, McHugo GJ, Green AI (2000). The effects of clozapine on alcohol and drug use disorders among patients with schizophrenia. Schizophr Bull.

[b12] Dusitanond P, Young WB (2009). Neuroleptics and migraine. Cent Nerv Syst Agents Med Chem.

[b13] Edlinger M, Hausmann A, Kemmler G (2005). Trends in the pharmacological treatment of patients with schizophrenia over a 12 year observation period. Schizophr Res.

[b14] Ekholm B, Ekholm A, Adolfsson R (2005). Evaluation of diagnostic procedures in Swedish patients with schizophrenia and related psychoses. Nord J Psychiatry.

[b15] Eriksson M, Holmgren L, Janlert U (2011). Large improvements in major cardiovascular risk factors in the population of northern Sweden: the MONICA study 1986–2009. J Intern Med.

[b16] Ettinger AB, Argoff CE (2007). Use of antiepileptic drugs for nonepileptic conditions: psychiatric disorders and chronic pain. Neurotherapeutics.

[b17] Galletti F, Cupini LM, Corbelli I, Calabresi P, Sarchielli P (2009). Pathophysiological basis of migraine prophylaxis. Prog Neurobiol.

[b18] Gerlach J (2002). Improving outcome in schizophrenia: the potential importance of EPS and neuroleptic dysphoria. Ann Clin Psychiatry.

[b19] Gillies D, Beck A, McCloud A, Rathbone J, Gilles D (2005). Benzodiazepines alone or in combination with antipsychotic drugs for acute psychosis. Cochrane Database Syst Rev.

[b20] Glick ID, Zaninelli R, Hsu C (2004). Patterns of concomitant psychotropic medication use during a 2-year study comparing clozapine and olanzapine for the prevention of suicidal behavior. J Clin Psychiatry.

[b21] Gray R, Gournay K (2000). What can we do about acute extrapyramidal symptoms?. J Psychiatr Ment Health Nurs.

[b22] Green AI, Noordsy DL, Brunette MF, O'Keefe C (2008). Substance abuse and schizophrenia: pharmacotherapeutic intervention. J Subst Abuse Treat.

[b23] Hamel E (2007). Serotonin and migraine: biology and clinical implications. Cephalalgia.

[b24] Haro JM, Salvador-Carulla L (2006). The SOHO (Schizophrenia Outpatient Health Outcome) study: implications for the treatment of schizophrenia. CNS Drugs.

[b25] Haspel T (1995). Beta-blockers and the treatment of aggression. Harv Rev Psychiatry.

[b26] Hayes PE, Schulz SC (1983). The use of beta-adrenergic blocking agents in anxiety disorders and schizophrenia. Pharmacotherapy.

[b27] Hedlund PB (2009). The 5-HT7 receptor and disorders of the nervous system: an overview. Psychopharmacology (Berl).

[b28] Huf G, Alexander J, Allen MH (2005). Haloperidol plus promethazine for psychosis induced aggression. Cochrane Database Syst Rev.

[b29] Jönsson EG, Edman-Ahlbom B, Sillén A (2006). Brain-derived neurotrophic factor gene (BDNF) variants and schizophrenia: an association study. Prog Neuropsychopharmacol Biol Psychiatry.

[b30] Jönsson EG, Saetre P, Vares M, Strålin P, Levander S, Lindström E (2011). Use of antipsychotics - an analysis of lifetime treatment in 66 patients with psychoses. Psychiatry Res.

[b31] Kane JM (2001). Extrapyramidal side effects are unacceptable. Eur Neuropsychopharmacol.

[b32] Kasckow JW, Zisook S (2008). Co-occurring depressive symptoms in the older patient with schizophrenia. Drugs Aging.

[b33] Kontis D, Theochari E, Kleisas S (2010). Doubtful association of antipsychotic polypharmacy and high dosage with cognition in chronic schizophrenia. Prog Neuropsychopharmacol Biol Psychiatry.

[b34] Koponen H, Alaraisanen A, Saari K (2008). Schizophrenia and sudden cardiac death: a review. Nord J Psychiatry.

[b35] Kornischka J, Cordes J, Agelink MW (2007). 40 years beta-adrenoceptor blockers in psychiatry. Fortschr Neurol Psychiatr.

[b36] Lawyer G, Nyman H, Agartz I (2006). Morphological correlates to cognitive dysfunction in schizophrenia as studied with Bayesian regression. BMC Psychiatry.

[b37] Leucht S, Kissling W, McGrath J (2007a). Lithium for schizophrenia. Cochrane Database Syst Rev.

[b38] Leucht S, Kissling W, McGrath J, White P (2007b). Carbamazepine for schizophrenia. Cochrane Database Syst Rev.

[b39] Levander S, Eberhard J, Lindström E (2007). Clinical decision-making during 5 years of antipsychotic treatment. Acta Psychiatr Scand Suppl.

[b40] Lima AR, Soares-Weiser K, Bacaltchuk J, Barnes TR (2002). Benzodiazepines for neuroleptic-induced acute akathisia. Cochrane Database Syst Rev.

[b41] Linde K, Rossnagel K (2004). Propranolol for migraine prophylaxis. Cochrane Database Syst Rev.

[b42] Lindström E, Farde L, Eberhard J, Haverkamp W (2005). QTc interval prolongation and antipsychotic drug treatments: focus on sertindole. Int J Neuropsychopharmacol.

[b43] Lipinski JF, Keck PE, McElroy SL (1988). Beta-adrenergic antagonists in psychosis: is improvement due to treatment of neuroleptic-induced akathisia?. J Clin Psychopharmacol.

[b44] Low NC, Du Fort GG, Cervantes P (2003). Prevalence, clinical correlates, and treatment of migraine in bipolar disorder. Headache.

[b45] Lubman DI, Berk M (2010). Pharmacotherapy for co-occurring alcohol and drug disorders in schizophrenia and bipolar disorder: where is the evidence?. Acta Neuropsychiatr.

[b46] Luo RD, Belleti DA, Tran D, Arcona S, Salen PN (2002). National prescribing patterns in the management of extrapyramidal symptoms among patients with schizophrenia. Int J Psychiatry Med.

[b47] Mackin P (2008). Cardiac side effects of psychiatric drugs. Hum Psychopharmacol.

[b48] Mallinger JB, Lamberti SJ (2007). Racial differences in the use of adjunctive psychotropic medications for patients with schizophrenia. J Ment Health Policy Econ.

[b49] McIntyre RS, Konarski JZ, Wilkins K, Bouffard B, Soczynska JK, Kennedy SH (2006). The prevalence and impact of migraine headache in bipolar disorder: results from the Canadian Community Health Survey. Headache.

[b50] Merikangas KR, Stevens DE (1997). Comorbidity of migraine and psychiatric disorders. Neurol Clin.

[b51] Meyer J (2007). Drug-drug interactions with antipsychotics. CNS Spectr.

[b52] Micallef J, Fakra E, Blin O (2006). Use of antidepressant drugs in schizophrenic patients with depression. Encephale.

[b53] Miller DD, McEvoy JP, Davis SM (2005). Clinical correlates of tardive dyskinesia in schizophrenia: baseline data from the CATIE schizophrenia trial. Schizophr Res.

[b54] Möller HJ (2004). Non-neuroleptic approaches to treating negative symptoms in schizophrenia. Eur Arch Psychiatry Clin Neurosci.

[b55] Morrison JR (1975). Catatonia: diagnosis and management. Hosp Community Psychiatry.

[b56] Novick D, Bousono M, Suarez D (2005). Use of concomitant medication with antipsychotic treatment in outpatients with schizophrenia: results from the European Schizophrenia Outpatients Health Outcomes (SOHO) study. Prog Neuropsychopharmacol Biol Psychiatry.

[b57] Ortiz A, Cervantes P, Zlotnik G (2010). Cross-prevalence of migraine and bipolar disorder. Bipolar Disord.

[b58] Pacher P, Kecskemeti V (2004). Cardiovascular side effects of new antidepressants and antipsychotics: new drugs, old concerns?. Curr Pharm Des.

[b59] Petrakis IL, Nich C, Ralevski E (2006). Psychotic spectrum disorders and alcohol abuse: a review of pharmacotherapeutic strategies and a report on the effectiveness of naltrexone and disulfiram. Schizophr Bull.

[b60] Pierre JM (2005). Extrapyramidal symptoms with atypical antipsychotics: incidence, prevention and management. Drug Saf.

[b61] Prakash R, Reed RM, Bass AD (1984). Combination of phenobarbital and haloperidol in resistant schizophrenia. J Clin Psychopharmacol.

[b62] Punay NC, Couch JR (2003). Antidepressants in the treatment of migraine headache. Curr Pain Headache Rep.

[b63] Pytliak M, Vargova V, Mechirova V, Felsoci M (2011). Serotonin receptors - from molecular biology to clinical applications. Physiol Res.

[b64] Rathbone J, Soares-Weiser K (2006). Anticholinergics for neuroleptic-induced acute akathisia. Cochrane Database Syst Rev.

[b65] Regier DA, Farmer ME, Rae DS (1990). Comorbidity of mental disorders with alcohol and other drug abuse. Results from the Epidemiologic Catchment Area (ECA) study. J Am Med Assoc.

[b66] Rosenberg SD, Goodman LA, Osher FC (2001). Prevalence of HIV, hepatitis B, and hepatitis C in people with severe mental illness. Am J Public Health.

[b67] Roth RM, Brunette MF, Green AI (2005). Treatment of substance use disorders in schizophrenia: a unifying neurobiological mechanism?. Curr Psychiatry Rep.

[b68] Sakai Y, Dobson C, Diksic M, Aube M, Hamel E (2008). Sumatriptan normalizes the migraine attack-related increase in brain serotonin synthesis. Neurology.

[b69] Schwarz C, Volz A, Li C, Leucht S (2008). Valproate for schizophrenia. Cochrane Database Syst Rev.

[b70] Shorter E (2009). The history of lithium therapy. Bipolar Disord.

[b71] Silberstein SD, Peres MF, Hopkins MM (2002). Olanzapine in the treatment of refractory migraine and chronic daily headache. Headache.

[b72] Silver H (2001). Fluvoxamine as an adjunctive agent in schizophrenia. CNS Drug Rev.

[b73] Silver H (2004). Selective serotonin re-uptake inhibitor augmentation in the treatment of negative symptoms of schizophrenia. Expert Opin Pharmacother.

[b74] Silver H, Chertkow Y, Weinreb O, Danovich L, Youdim M (2009). Multifunctional pharmacotherapy: what can we learn from study of selective serotonin reuptake inhibitor augmentation of antipsychotics in negative-symptom schizophrenia?. Neurotherapeutics.

[b75] Soares KV, McGrath JJ (2000). Anticholinergic medication for neuroleptic-induced tardive dyskinesia. Cochrane Database Syst Rev.

[b76] Stimmel GL (1996). Benzodiazepines in schizophrenia. Pharmacotherapy.

[b77] Suzuki A, Yasui-Furukori N, Mihara K (2003). Histamine H1-receptor antagonists, promethazine and homochlorcyclizine, increase the steady-state plasma concentrations of haloperidol and reduced haloperidol. Ther Drug Monit.

[b78] Swartz MS, Wagner HR, Swanson JW (2006). Substance use in persons with schizophrenia: baseline prevalence and correlates from the NIMH CATIE study. J Nerv Ment Dis.

[b79] Vares M, Ekholm A, Sedvall GC, Hall H, Jönsson EG (2006). Characterisation of patients with schizophrenia and related psychosis: evaluation of different diagnostic procedures. Psychopathology.

[b80] Volz A, Khorsand V, Gillies D, Leucht S (2007). Benzodiazepines for schizophrenia. Cochrane Database Syst Rev.

[b81] Williams CL, Johnstone BM, Kesterson JG, Javor KA, Schmetzer AD (1999). Evaluation of antipsychotic and concomitant medication use patterns in patients with schizophrenia. Med Care.

[b82] World Health Organization (2007). Collaborating Centre for Drug Statistics Methodology. http://www.whocc.no/atcddd/.

[b83] Zaremba PD, Bialek M, Blaszczyk B, Cioczek P, Czuczwar SJ (2006). Non-epilepsy uses of antiepilepsy drugs. Pharmacol Rep.

[b84] Zimmet SV, Strous RD, Burgess ES, Kohnstamm S, Green AI (2000). Effects of clozapine on substance use in patients with schizophrenia and schizoaffective disorder: a retrospective survey. J Clin Psychopharmacol.

[b85] Zink M, Englisch S, Meyer-Lindenberg A (2010). Polypharmacy in schizophrenia. Curr Opin Psychiatry.

